# Supramolecular Self-Assembly of Atomically Precise Silver Nanoclusters with Chiral Peptide for Temperature Sensing and Detection of Arginine

**DOI:** 10.3390/nano12030424

**Published:** 2022-01-27

**Authors:** Wenjuan Wang, Zhi Wang, Di Sun, Shulin Li, Quanhua Deng, Xia Xin

**Affiliations:** National Engineering Research Center for Colloidal Materials, Key Laboratory of Colloid and Interface Chemistry (Ministry of Education), School of Chemistry and Chemical Engineering, Shandong University, Jinan 250100, China; wenjuanwang@mail.sdu.edu.cn (W.W.); zwang@mail.sdu.edu.cn (Z.W.); dsun@sdu.edu.cn (D.S.); 202032370@mail.sdu.edu.cn (S.L.); qhdeng@sdu.edu.cn (Q.D.)

**Keywords:** supramolecular self-assembly, metal nanoclusters, peptide, aggregation-induced emission, chirality

## Abstract

Metal nanoclusters (NCs) as a new type of fluorescent material have attracted great interest due to their good biocompatibilities and outstanding optical properties. However, most of the studies on metal NCs focus on the synthesis, atomic or molecular assembly, whereas metal NCs ability to self-assemble to higher-level hierarchical nanomaterials through supramolecular interactions has rarely been reported. Herein, we investigate atomic precise silver NCs (Ag_9_-NCs, [Ag_9_(mba)_9_], where H_2_mba = 2-mercaptobenzoic acid) and peptide DD-5 were used to induce self-assembly, which can trigger an aggregation-induced luminescence (AIE) effect of Ag_9_-NCs through non-covalent forces (H-bond, π–π stacking) and argentophilic interactions [Ag(I)–Ag(I)]. The large Stokes shift (~140 nm) and the microsecond fluorescence lifetime (6.1 μs) indicate that Ag_9_-NCs/DD-5 hydrogel is phosphor. At the same time, the chirality of the peptide was successfully transferred to the achiral Ag_9_-NCs because of the supramolecular self-assembly, and the Ag_9_-NCs/DD-5 hydrogel also has good circularly polarized luminescence (CPL) properties. In addition, Ag_9_-NCs/DD-5 luminescent hydrogel is selective and sensitive to the detection of small biological molecule arginine. This work shows that DD-5 successfully induces the self-assembly of Ag_9_-NCs to obtain high luminescent gel, which maybe become a candidate material in the fields of sensors and biological sciences.

## 1. Introduction

As a bridge between atom and nanoparticle, metal nanoclusters (metal NCs, mainly Au, Ag and Cu) which are composed of a few to hundreds of mental atoms and covered with organic ligands on the surface of the metal core, have been receiving extensive attention due to their important roles in the fields of catalysis, sensing, electrochemistry, energy transfer and biomedicine [1,2,3,4,5]. Among various applications, metal nanoclusters are widely used as sensors because of their sensitive responses to temperature, pH value, metal ions and small biological molecules [6,7,8,9,10]. For example, Hu et al. utilized Cu-MOF as a precursor to prepare highly stable Cu NCs, which can be used to construct a pH ratiometric fluorescence sensor to monitor pH of microorganism [8]. Kailasa et al. found that the addition of La^3+^ can significantly enhance the fluorescence emission of BSA-AuNCs, and constructed an La^3+^ ion-BSA-AuNCs fluorescence sensor, thus realizing the detection of four divalent metal ions (Hg^2+^, Cy^2+^, Pb^2+^ and Cd^2+^) [9].

In particular, the biocompatibility and fluorescent properties are potentially useful in biological systems of Ag NCs, making them a research hotspot [11]. However, the current research on Ag NCs is mainly focused on the synthesis and atomic or molecular assembly, while the nanostructures obtained by the means of supramolecular self-assembly are rarely reported [12,13]. This may be due to the small size of the Ag NCs, and to the higher surface energy inducing further growth of Ag NCs into larger Ag nanoparticles, which is not conducive to the progress of self-assembly. Therefore, in order to solve this problem, the subtle non-covalent interactions (H-bond, π–π stacking, van der Waals forces, electrostatic interactions and hydrophobic interaction) should be controlled between Ag NCs by adding small molecules through supramolecular self-assembly to obtain ordered aggregates.

As a natural biomolecule, peptides are usually composed of less than 50 amino acids, are easy to manipulate and synthesize, and can self-assemble into various ordered aggregates, such as nanotubes, nanofibers, nanovesicles, nanobelts and hydrogel [14,15,16,17,18]. However, in order to overcome the inherent limitations of single-component materials and make multi-component materials more widely used, peptides can be co-assembled with a variety of functional molecules to obtain supramolecular materials [19,20,21]. For example, Gazit et al., through the co-assembly between Fmoc-RGD and chitosan, obtained a hydrogel with stronger mechanical properties and stronger durability than Fmoc-RGD self-assembled hydrogel, which has a wide range of applications in the field of cell culture scaffolds [22]. Xu et al. obtained the hydrogel of nanofibers through coordination, H-bond and hydrophobic interaction between hairpin peptides and copper ions, and the nanofibers can be utilized as templates for the synthesis of long, ultrathin CuS nanowires which have near-infrared (NIR) laser-induced thermal effect [23]. Therefore, using peptides to induce the self-assembly of Ag NCs can not only improve the biocompatibility of multi-component materials, but also expand the range of application for metal NCs-based materials [6,7,24].

**Scheme 1:**
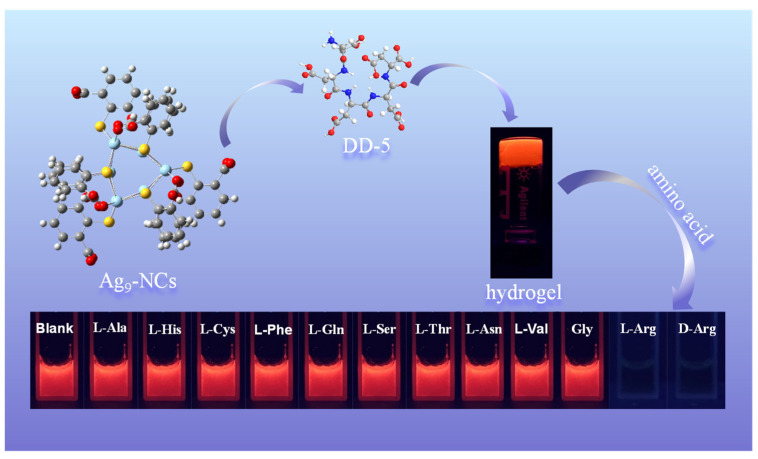
Schematic illustration of the self-assembly of the Ag_9_-NCs driven by DD-5 and their application in the detect of Arginine.

In this work, we used water-soluble, atomically precise Ag NCs (Ag_9_-NCs, [Ag_9_(mba)_9_], where H_2_mba = 2-mercaptobenzoic acid) to interact with the peptide DD-5 to construct luminescent hydrogel. The Ag_9_-NCs/DD-5 hydrogel was realized through non-covalent forces (H-bond, π–π stacking) and argentophilic interactions [Ag(I)–Ag(I)], and phosphorescent emission was obtained through aggregation-induced luminescence (AIE). The Ag_9_-NCs/DD-5 xerogel has good application in temperature sensing and the orange-red emission of the hydrogel can be used to detect arginine (Scheme 1). This work provides a new example for the construction of metal NC-peptide complexes through non-covalent bonds used in the fields of temperature fluorescence sensing and biological detection.

## 2. Materials and Methods

### 2.1. Materials

**Figure 1:**
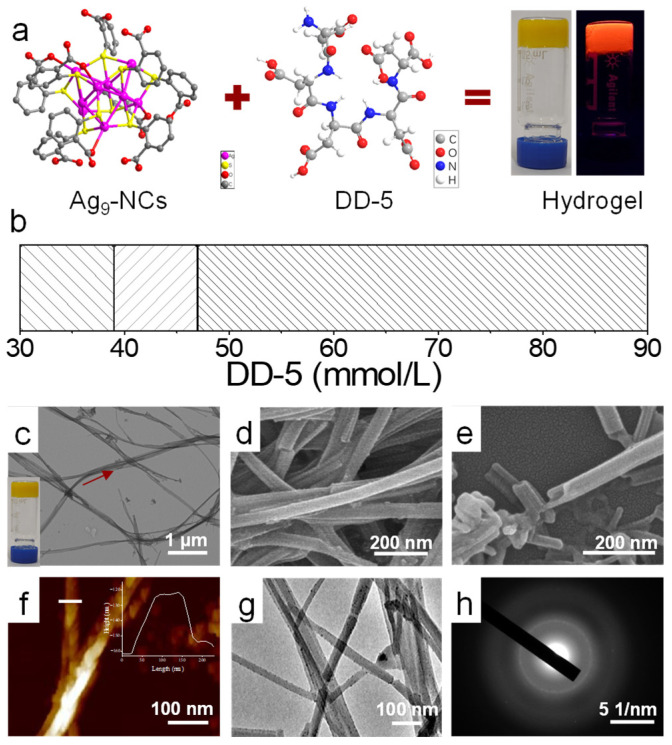
Self-assembly phase behavior and detailed characterization of 5 mM Ag_9_-NCs/70 mM DD-5 hydrogel. (**a**) The structure of Ag_9_-NCs and DD-5, the schematic diagram of the formation of the core sample hydrogel; (**b**) the concentration of Ag_9_-NCs was fixed at 5 mM, and the concentration of DD-5 was changed to obtain a phase diagram; (**c**) TEM, the inset is a visual diagram of the sample; (**d**,**e**) SEM; (**f**) AFM, inset: a plan view of the height extending from the indicator line; (**g**) HR-TEM; (**h**) SAED pattern of HR-TEM.

The synthesis, purification and detailed characterization of Ag_9_-NCs can be found in literature [6,25]. DD-5 was purchased from GL Biochem Ltd. (Shanghai, China) and used without further purification. It was polymerized by five aspartic acids and its relative molecular mass was 593.46 g mol^−1^. The molecular structure of DD-5 is shown in Figure 1a. L-Arginine (L-Arg), D-Arginine (D-Arg), L-alanine (L-Ala), L-histidine (L-His), L-cysteine (L-Cys), L-phenylalanine (L-Phe), L-tyrosine (L-Tyr), L-asparagine (L-Asn), L-valine (L-Val) and glycine (Gly) were purchased from Sinopharm Chemical Reagent Co., Ltd, (Shanghai, China). L-Glutamine (L-Glu) and L-serine (L-Ser) were purchased from Sigma-Aldrich (St. Louis, MO, USA). Ultrapure water used in the experiments with a resistivity of 18.25 MΩ cm^−1^ was obtained using a UPH-IV ultrapure water purifier (Shanghai Youpu Industry Company Ltd., Shanghai, China).

### 2.2. Self-Assembly of Ag9-NCs/DD-5 Hydrogel

In this typical experiment, we added 20.0 mg DD-5 to 355 μL ultrapure water and stirred, then added 145 μL Ag_9_-NCs solution (15.87 mM) and stirred. The hydrogel was successfully prepared after 8 h of constant temperature (20 °C) in a thermostat. The hydrogel was lyophilized in a vacuum extractor at −60 °C for 5 days to collect the orange-yellow powder.

### 2.3. The Detection of L-Arg and D-Arg

Amino acids were added to the solution of DD-5 and Ag_9_-NCs to ensure that the final concentration of amino acids was 100 mM. After incubating in 20 °C incubator for 8 h, we performed a fluorescence test to study the selectivity of the hydrogel toward L-Arg and D-Arg, because both D-Arg and L-Arg can completely quench the fluorescence of nanotubes. We took L-Arg as an example to investigate in detail: we added different concentrations of L-Arg to the formed 100 μL of Ag_9_-NCs/DD-5 hydrogel, and placed it in a thermostat at 20 °C for 8 h. We used a triangular cuvette with a capacity of 2 mL for fluorescence detection to obtain concentration change spectra.

### 2.4. Characterizations

A copper mesh was inserted into the gel to obtain a sample, and after drying under an IR lamp for 45 min, transmission electron microscopy (TEM) images were observed under a JCR-100CX II (JEOL, Tokyo, Japan) microscope. Field emission scanning electron microscopy (FE-SEM) observations were carried out on a Hitachi SU8010 (Hitachi, Tokyo, Japan) under 10 kV. High-resolution TEM (HRTEM) images and selected area electron diffraction (SAED) were recorded by HR-JEOL 2100 (JEOL, Tokyo, Japan) system with an accelerating voltage of 200 kV. Atomic force microscope (AFM) tapping mode measurements were performed on Bruke Bioscope Resolve. UV-vis data were recorded on a Shimadzu UV-2600 spectrophotometer (Shimadzu, Kyoto, Japan). Fluorescence data were tested on a LS-55 spectrofluorometer (PerkinElmer, Waltham, MA, USA) and an Edinburgh Instruments FLS920 luminescence spectrometer (xenon lamp, 450 W) (Edinburgh Instruments Ltd., Livingston, UK). Fourier transform infrared (FT-IR) spectra in KBr wafer were recorded on a VERTEX-70/70v spectrophotometer (Bruker, Billerica, MA, USA). CD spectra were taken on J-810 Spectra Manager system (ChirascanV 100). Small-angle X-ray spectroscopy (SAXS) measurements were performed using an Anton-Paar SAX Sess mc^2^ system (Anton Paar, Graz, Austria) with nickel-filtered Cu Kα radiation (1.54 Å) operating at 50 kV and 40 mA. X-ray diffraction (XRD) patterns were taken on a D8 ADVANCE (Bruker, Germany) diffractometer equipped with Cu Kα radiation and a graphite monochromator. X-ray photoelectron spectroscope (XPS) date were collected by an X-ray photoelectron spectrometer (ESCALAB 250, Thermo Fisher Scientific, Waltham, MA, USA) with a monochromatized AI Kα X-ray source (1486.71 eV). The rheological measurements were carried out on an MARS60 rheometer (Thermo Fisher Scientific, Waltham, MA, USA) with a cone−plate system. Before the frequency sweep, an amplitude sweep at a fixed frequency of 1 Hz was carried out to ensure that the selected stress was in the linear viscoelastic region. The frequency sweep was carried out from 0.01 to 100 Hz at a fixed stress of 10 Pa. The variable temperature spectrum is recorded in the UV-Vis-microspectrophotometer (20/30 PV^TM^, Craic Technologies, San Dimas, CA, United States). Thermogravimetric analyses (TGA) were performed under a nitrogen atmosphere at 25–1000 °C with a heating speed of 10 °C min^−1^ on a TA SDT Q600 thermal analyzer (TA Instruments, New Castle, DE, USA).

## 3. Results

### 3.1. Self-Assembly of Ag_9_-NCs/DD-5 Hydrogel

The average diameter of Ag_9_-NCs in the aqueous solution was about 1.4 ± 0.5 nm, and they were in a non-fluorescent state (Figure S1a). We wonder whether DD-5 peptides, which are non-fluorescent at room temperature, can be used to induce the AIE effect of Ag_9_-NCs through a self-assembly strategy (Figure S1b). The schematic diagram of the core sample self-assembly is shown in Figure 1a. The concentration of Ag_9_-NCs was fixed at 5 mM, the concentration of DD-5 was changed, and after incubation for 8 h, the phase diagram was obtained (Figure 1b). Under 365 nm UV light, the solution state (30~38 mM DD-5) did not emit fluorescence, while the precipitated state (38~47 mM DD-5) and the hydrogel state (47~90 mM DD-5) both emitted orange-red fluorescence (Figure S1c), which implied that as the concentration of DD-5 increases, Ag_9_-NCs gelation restricted the rotation or vibration of the Ag_9_-NCs ligand on the spatial scale, enhancing AIE and emitted fluorescence.

From the TEM and SEM images, it was found that the precipitate was a solid nanorod structure, and the hydrogel was hollow nanotube with a little spiral (Figure S2). When *c*_DD-5_ = 70 mM, it is located in the center of the hydrogel and its morphology is better (Figure S2), so it is defined as the core sample for follow-up research. The microstructures within the hydrogel were characterized in detail by imaging studies. TEM shows that the fibers formed in 5 mM Ag_9_-NCs/70 mM DD-5 hydrogel were about 30–50 nm in width and 5–20 μm micrometers in length (Figure 1c,d) and the inside of the fiber had lower contrast than the edge (Figure 1c,g). The fracture surface observed by SEM (Figure 1d,e) indicated that the fibers were hollow nanotube structures. AFM image shows that the thickness of the nanotube was 40 nm, which was basically the same as the width of nanotubes (Figure 1f). In addition, SAED results in Figure 1h indicate that the nanotubes were a polycrystalline structure.

### 3.2. Fluorescence and Chirality of Ag_9_-NCs/DD-5 Hydrogel

**Figure 2:**
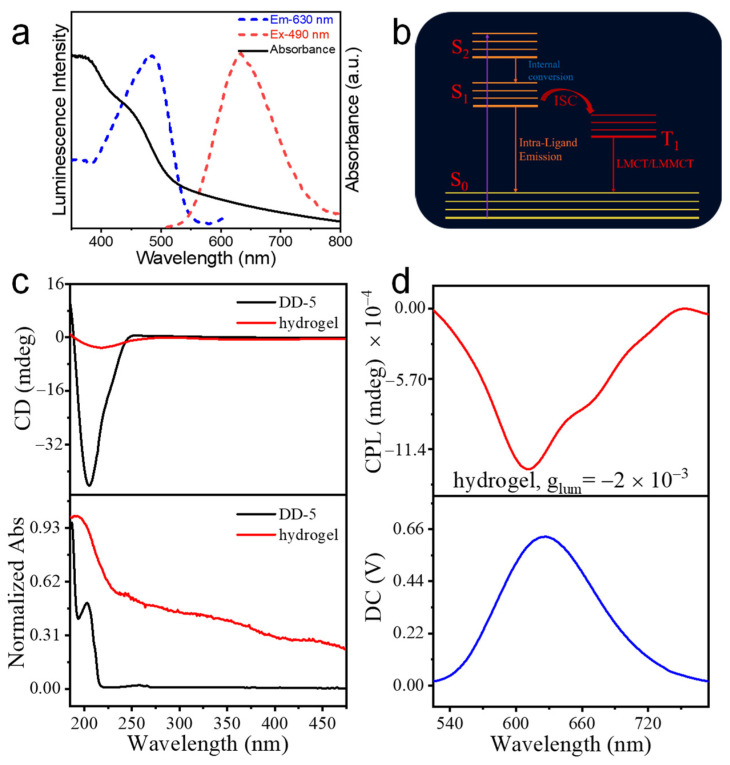
Study on the optical and chiral behavior of Ag_9_-NCs/DD-5 hydrogel. (**a**) Excitation (blue dotted line, λ_e__m_ = 630 nm), emission (red dotted line, λ_ex_ = 490 nm) spectra and UV-vis absorption (black line); (**b**) schematic diagram of the excite-state relaxation dynamics of the nanotubes; (**c**) CD spectrum of pure DD-5 and hydrogel, the bottom are the UV absorption spectra of pure DD-5 and hydrogel; (**d**) CPL spectrum of hydrogel, the DC value in the bottom spectrum stands for fluorescence intensity of hydrogel.

As metal NCs are known as potential AIE molecules, we next explored the fluorescent properties of Ag_9_-NCs/DD-5 hydrogel. In the solution state of Ag_9_-NCs, due to the free vibration or rotation of the ligand mba^2−^, the non-radiative inactivation channel is opened and the fluorescence disappears. Therefore, Ag_9_-NCs have a shorter fluorescence lifetime (~3.277 ns) (Figure S3a, Table S1). However, under the excitation wavelength of 490 nm, the quantum yield of the Ag_9_-NCs/DD-5 hydrogel is 8.11% with a microsecond fluorescence lifetime (~6.10 μs), and the emission wavelength is located at 630 nm with a large Stokes shift (~140 nm) (Figure 2a and Figure S3b, Table S2), indicating that it is essentially a phosphor.

There are several reasons to prove that the system is phosphorescence produced by triple transitions: (i) the optimal excitation peak for Ag_9_-NCs/DD-5 hydrogel is located at approximately 490 nm, overlapping with the absorption peak of the charge transfer from the ligand to the metal of Ag_9_-NCs caused by the addition of DD-5 (Figure 2a), inducing a phosphor with long lifetime emission [26]; (ii) the aromatic carboxyl group at room temperature is phosphorescence (RTP) molecule, the energy levels of singlet and triplet of H_2_mba are very similar, and after the introduction of DD-5 through non-covalent interactions the amount of charge transfer from ligand-to-metal charge transfer (LMCT) increases, which makes intersystem crossing (ISC) prone to occur [27]; (iii) charge transfer can be caused by argentophilic interactions, which can also be attributed to the triplet state of argentophilic interactions with ligand-metal-mental charge transfer (LMMCT) [28,29]. Thus, it can be concluded that after DD-5 is added, DD-5 combines with Ag_9_-NCs through non-covalent interactions, which limits the intramolecular vibration and rotation of the Ag_9_-NCs ligand mba^2−^, reducing the non-radiative loss of triplet excitons, and promotes phosphorescence emission (Figure 2b).

Based on the chirality of the peptide DD-5, we next examined the chirality of Ag_9_-NCs/DD-5 hydrogel. From the CD spectrum (Figure 2c), it is found that DD-5 has a strong negative Cotton effect at 204 nm, while Ag_9_-NCs/DD-5 hydrogel has a relatively weak negative Cotton effect at 217 nm, which indicates that the configuration after assembly has changed [30]. At the same time, the red shift 13 nm indicates that the chirality of DD-5 was successfully transferred to the assembly, and supramolecular chirality is obtained. Due to Ag_9_-NCs/DD-5 hydrogel having good luminescence performance and chirality, the circularly polarized luminescence (CPL) performance of the assembly is investigated. Generally speaking, the strength of CPL can be evaluated by the luminescence dissymmetry factor (g_lum_), which is defined as g_lum_ = 2 × (*I_L_ − I_R_*)/(*I_L_* + *I_R_*), where *I_L_* and *I_R_* refer to the intensity of left- and right-hand CPL, respectively. From the CPL spectrum (Figure 2d), the assembly has good CPL performance and asymmetry factor glum is −2.0 × 10^−3^, indicating that Ag_9_-NCs/DD-5 hydrogel with a negative Cotton effect displayed left-handed CPL.

### 3.3. Structure and Mechanism Analysis of the Hydrogel

**Figure 3:**
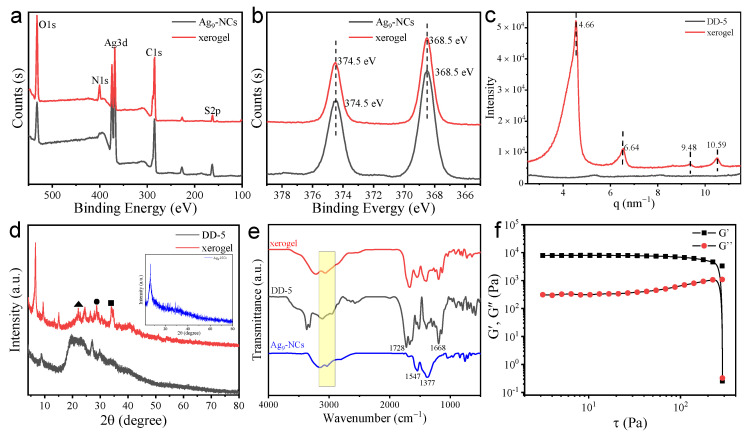
The structure characterization results of Ag_9_-NCs/DD-5 xerogel (or hydrogel). (**a**) XPS spectra of lyophilized Ag_9_-NCs and xerogel; (**b**) High-resolution XPS spectra of Ag element; (**c**) SAXS results of DD-5 and xerogel; (**d**) XRD results of DD-5 and xerogel, the inset shows the result of lyophilized Ag_9_-NCs; (**e**) FT-IR spectra of a lyophilized Ag_9_-NCs, DD-5 and xerogel; (**f**) stress sweep test at *f* = 1 Hz for the hydrogel Study on the optical and chiral behavior of Ag_9_-NCs/DD-5 hydrogel.

To dissect the self-assembly mechanism of Ag_9_-NCs/DD-5 hydrogels, it is essential to analyze the composition of supramolecular assembly. The peak splitting results of C_1s_, N_1s_, O_1s,_ S_2p_ and Ag_3d_ for Ag_9_-NCs/DD-5 xerogel in XPS indicate that the hydrogel is formed by Ag_9_-NCs and DD-5 (Figure 3a and Figure S4). Moreover, Ag_9_-NCs/DD-5 xerogel and Ag_9_-NCs powder have the similar signals in Ag 3d_5/2_ and Ag 3d_3/2_, indicating that Ag_9_-NCs do not undergo chemical or structural conversions during gelation (Figure 3b). In order to understand the deposition pattern and spatial structure of the hydrogel, SAXS and XRD were carried out. For the SAXS result of Ag_9_-NCs/DD-5 xerogel, four diffraction peaks are found at *q* = 10.59, 9.48, 6.64 and 4.66 nm^−1^ with a scattering factor *q* ratio of 1:√2:2:√5, which is a typical tetragonal phase stack (Figure 3c) [31]. In addition, the smallest repeating unit of its aggregate *d* = 1.35 nm, which is equivalent to the size of Ag_9_-NCs. Considering the length of the Ag_9_-NCs ligand mba^2−^ ligand (~6.5 Å) and the length of DD-5 molecule, the smallest repeating unit of 1.35 nm indicates a strong crossover between adjacent Ag_9_-NCs and DD-5, supporting the π–π stacking form of Ag_9_-NCs.

As shown in the XRD spectrum (Figure 3d), the Ag_9_-NCs/DD-5 xerogel has several relatively significant peaks were recorded in the range of 20–45°. The diffraction peaks at 2θ = 22.14°, 28.62° and 34.10° correspond to π–π stacking (peak at ▲ 4.0 Å), Ag-Ag (peak at ● 3.1 Å), and Ag-S (peak at ■ 2.6 Å) and other possible interplanar distances [32,33], which indicate that the presence of π-π stacking and Ag–Ag interactions contribute to an ordered arrangement in the assembled hydrogel. In contrast, the XRD of lyophilized Ag_9_-NCs solution and pure DD-5 only showed a diffuse reflection peak, indicating that they are of an amorphous nature. Based on the above data, we can conclude that the self-assembly process is closely related to the non-covalent interactions (H-bond, π–π stacking) between Ag_9_-NCs and DD-5 and argentophilic interactions [Ag(I)–Ag(I)] between Ag_9_-NCs, and finally highly ordered fluorescent nanotubes are obtained.

FT-IR was performed to explore the non-covalent forces between Ag_9_-NCs and DD-5 in the self-assembly (Figure 3d). For pure Ag_9_-NCs powder, 1537 and 1377 cm^−1^ are assigned to the antisymmetric and symmetric stretching vibrations of C=O in the ligand mba^2−^ (Figure 3e) [34]. For pure DD-5 powder, the peak at 1410–1260 cm^−1^ is assigned to the in-plane curvature of the free carboxyl group, the peaks at 1668 and 1728 cm^−1^ belong to the amide I band, which is attributed to the stretching vibration of the peptide backbone, and the peak at 3370–3320 cm^−1^ is attributed to the stretching vibration of N-H. In Ag_9_-NCs/DD-5 xerogel, it was found that the C=O of the Ag_9_-NCs ligand showed a significant red shift, the DD-5 amide I band peak disappeared and the free carboxyl group in-plane curvature peak weakened or even disappeared, indicating that there are hydrogen bonds between Ag_9_-NCs and DD-5. Moreover, the widening of the stretching vibration absorption band of 3200–3000 cm^−1^ belongs to –OH, which also proves the formation of hydrogen bonds in this system [35].

The rheological characteristics are of great significance to supramolecular materials in the gel state, so we next tested the rheological properties of Ag_9_-NCs/DD-5 hydrogel to evaluate the mechanical properties of the hydrogel [36]. In the stress scan, the storage modulus (G’) is much larger than the loss modulus (G”), indicating that the hydrogel exhibits solid-like nature (Figure 3f). The yield stress corresponds to the transition from gel to fluid, that is, the network structure of the gel is destroyed [37], and Ag_9_-NCs/DD-5 hydrogel basically remains unchanged before reaching the yield stress of 285 Pa, indicating that the formed hydrogel has high rigidity and resistance to damage ability. In the frequency sweep experiment, the storage modulus (G’) and loss modulus (G”) of the hydrogel remained basically unchanged, and G’ (2300 Pa) was much larger than G” (115 Pa), indicating that Ag_9_-NCs/DD-5 hydrogel has a good mechanical strength (Figure S5a).

TGA was performed to explore the thermal stability of the hydrogel (Figure S5b). The weight loss of the xerogel sample before 100 °C can be attributed to water loss. The weight loss at 200–285 °C is attributed to the vaporization of DD-5 carbon and the removal of oxidized functional groups (the first stage decomposition temperature of pure DD-5 at 174 °C), together with the decomposition of H_2_mba (the decomposition temperature of lyophilized Ag_9_-NCs ligand H_2_mba is 181 °C). Compared with the original DD-5 and Ag_9_-NCs, the decomposition temperature of Ag_9_-NCs/DD-5 xerogel increased slightly (200 °C), indicating that the thermal stability of the xerogel was further improved.

Based on the above results, it can be concluded that DD-5 and Ag_9_-NCs were successfully co-assembled to obtain highly stable hydrogel. The hydrogel consists of highly ordered hollow nanotubes with a little spiral forming a stable three-dimensional network. We can propose the formation mechanism of nanotubes: (i) After the addition of DD-5, the rotational vibration of the mba^2−^ is restricted by H-bond formed which was surmised as being further divided in several parts, such as –COO^−^ of mba^2−^ with –OH (or –NH–) of DD-5, and/or –OH of protonated mba^2−^ (H_2_mba) with –COO^−^ of DD-5 and so on. (ii) The adjacent components with Ag(I)-rich surface led to the strong argentophilic interactions between Ag_9_-NCs which induces the formation of highly oriented nanotubes. The π–π stacking of adjacent ligands also contributes to the formation of spiral nanotubes. Therefore, the assembly process of hollow spiral nanotubes is controlled by inter-ligand non-covalent interactions (e.g., H-bond, π–π stacking) and argentophilic interactions between Ag_9_-NCs.

### 3.4. Kinetic Tracing of the Formation of Ag_9_-NCs/DD-5 Hydrogel

**Figure 4:**
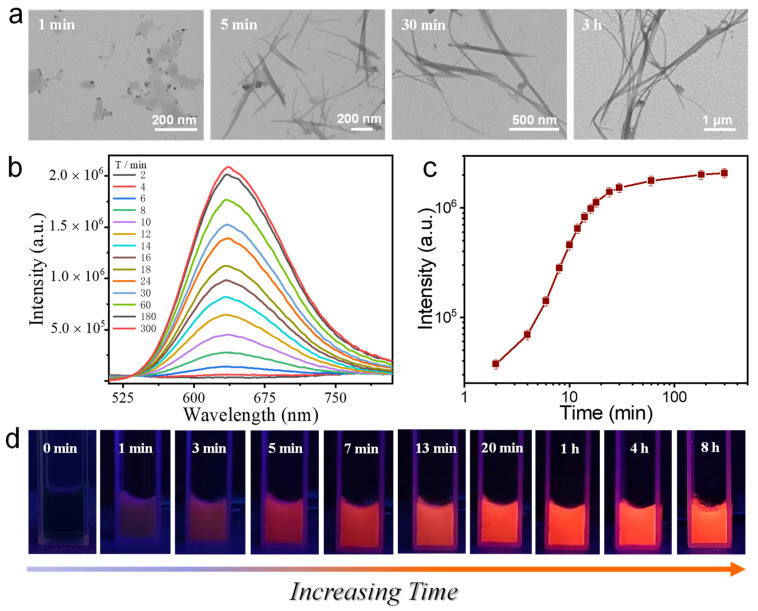
Ag_9_-NCs/DD-5 hydrogel kinetic tacking. (**a**) TEM images of hydrogel changing over time; (**b**) Time-varying PL spectrum of hydrogel under excitation at 490 nm; (**c**) PL intensity graph at *λ* = 630 nm (*λ_ex_* = 490 nm); (**d**) Photographs of the formation process of the hydrogel irradiated with 365 nm UV light.

In order to explore the relationship between the gelation process and fluorescent properties of Ag_9_-NCs/DD-5 hydrogel, we followed the kinetic tracking of the self-assembly (Figure 4d). TEM showed the evolution of the morphology during the gelation (Figure 4a). At 1 min, the self-assembled body is a small particle of Ag_9_-NCs combined with DD-5, there is no fluorescence emission (Figure 4a,c); The small particles self-assemble into a hollow short rod structure with a length of about 500 nm in 5 min, and weak fluorescence appears at this time; at 30 min, the hollow short rod structure further grows into nanotubes with a length of about 1 μm, and the fluorescence of the nanotubes is obviously enhanced; nanotubes with a little spiral structure are about 2 μm in length at 3 h, and the fluorescence is continually increased compared with that at 30 min, but it is not much different from the final state fluorescence. Therefore, we believe that fluorescence emission is inseparable from the gelation process, that is, the increase in fluorescence intensity is caused by the transition from loose assembly to tightly packed spiral nanotubes. Based on the above results, we believe that in the presence of argentophilic interactions and non-covalent forces (H-bond, π–π stacking), a tightly ordered assembly structure is formed which spatially limits the vibration and rotation of Ag_9_-NCs ligands, thus triggers the AIE effect of the Ag_9_-NCs and induces fluorescence. That is, as the order of the assembled structure of Ag_9_-NCs/DD-5 hydrogel increases, the fluorescence gradually increases.

### 3.5. Temperature Sensing

Optical sensors have the advantages of wireless operation and imaging in harsh environments. Moreover, it is very important to realize temperature monitoring in scientific production, which also promotes the development of temperature sensing. The ultra-small size, good biocompatibility and colloidal stability of metal NCs are considered to be good materials for the development of high-intensity fluorescence thermometers. However, temperature sensors based on metal NCs are mainly concentrated in solution and gel states, while solid-state temperature sensors are rarely explored. In addition, most solid-state phosphorescent temperature sensing materials have poor photoluminescence capabilities at high temperatures, and the fluorescence generally disappears when the temperature is higher than 100 °C [38,39,40]. Therefore, it is particularly important to develop a solid material with good photoluminescence ability at high temperature. Based on this, we freeze-dried the hydrogel to obtain phosphorescent xerogel, and explored the luminescence of the xerogel with temperature changes in detail.

**Figure 5:**
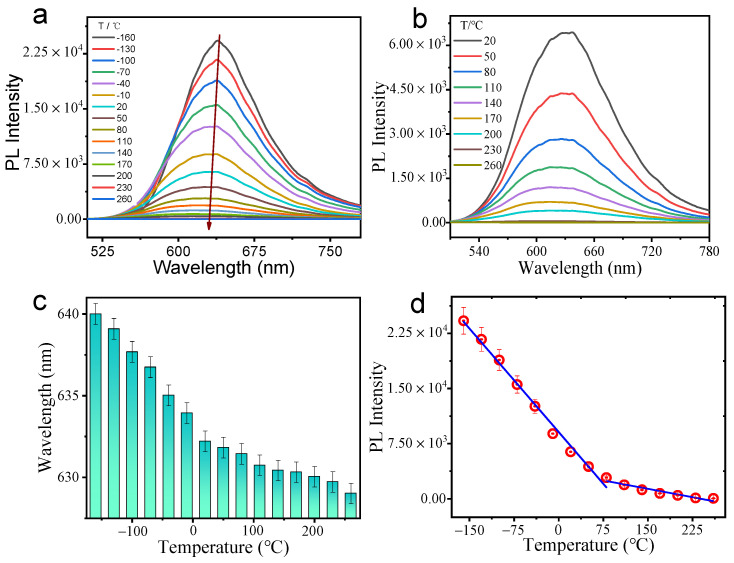
Fluorescence change of xerogel with temperature. (**a,b**) Fluorescence spectra with increasing temperature; (**c**) PL peak position changes with increasing temperature; (**d**) The corresponding PL intensity under different temperature.

As the temperature rises from −160 °C to 260 °C, the PL intensity of Ag_9_-NCs/DD-5 xerogel continues to decrease and the fluorescence peak position also appears slightly blue-shifted (Figure 5a–c). The reasons for this change are as follows: (i) as the temperature increases, the oxygen molecules collide with their luminescent center; (ii) the high temperature weakens the Ag-S bond between the H_2_mba and the sliver core, resulting in a decrease in the charge from the ligand to the sliver core of Ag_9_-NCs; (iii) as the temperature rises, non-covalent interactions (H-bond, π–π stacking) gradually weaken, resulting in a compact network being stretched, the rotation limit of the mba^2−^ ligand is reduced, non-radiative relaxation is prone to occur; and (iv) as the temperature increases, the conformation of DD-5 changes, causing Ag_9_-NCs to be directly exposed to the air and oxidized to larger silver nanoparticles at high temperatures. All these factors lead to the decrease of fluorescence intensity and blue shift of fluorescence peak. Figure 5d shows the change in PL intensity over the entire temperature range, which can be described by two linear parts with an inflection point at 80 °C (Figure 5d). The xerogel satisfies a good linear relationship in the low temperature range from −160 °C to 80 °C and the high temperature range from 80 °C to 260 °C, which will have a wide range of applications in the field of both low and high temperature fluorescence sensing.

### 3.6. The Detection of L-Arg and D-Arg

**Figure 6:**
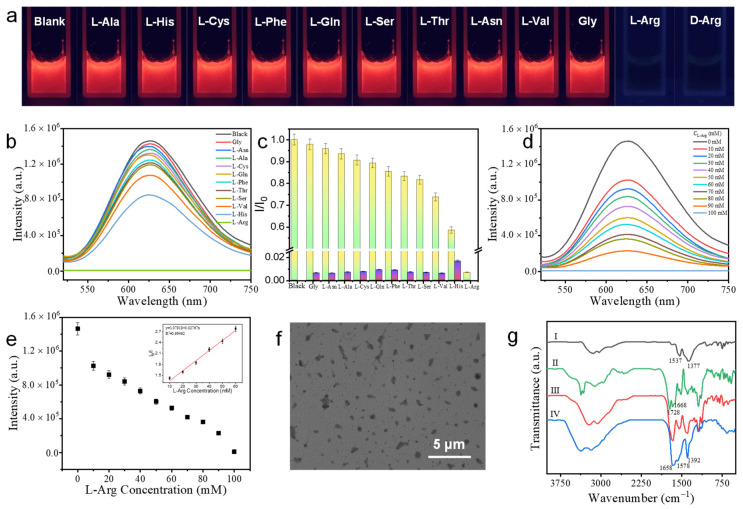
Detection of arginine by hydrogel. (**a**) Photographs of the samples observed under 365 nm UV lamp with the addition of amino acids; (**b**) PL spectra of nanotubes with the addition of amino acids; (**c**) Selectivity of nanotubes toward L-Arg, the lighter color is the fluorescence intensity after adding different kinds of amino acids, the darker color is the fluorescence intensity after adding L-Arg to the above sample; (**d**,**e**) Concentration-dependent fluorescence spectra and fluorescence intensity with the addition of different contents of L-Arg, inset: Stern-Volmer quenching curve of the fluorescence intensity of the hydrogel at 630 nm against L-Arg concentration; (**f**) TEM image of L-Arg added to Ag_9_-NCs/DD-5 hydrogel; (**g**) FT-IR spectrum, where I represents lyophilized Ag_9_-NCs; II represents DD-5; III represents Ag_9_-NCs/DD-5 xerogel; IV represents freeze-dried sample after adding L-Arg to Ag_9_-NCs/DD-5 hydrogel.

It is well-known that amino acids play an important role in the human body. Among various amino acids, Arg can stimulate the secretion of hormones such as insulin, growth hormone, glucagon and prolactin. At the same time, Arg content is also one of the key parameters for evaluating the pathophysiology of hyperammonemia and astrocytes cultured with aggregated nerve cells [41,42,43]. Therefore, it is very important for human health to detect Arg with high selectivity and sensitivity. Fortunately, Ag_9_-NCs/DD-5 hydrogel with good biocompatibility can be used as a biosensor to detect L-Arg and D-Arg. It can be found that the addition of L-Arg and D-Arg can completely quench the fluorescence of Ag_9_-NCs/DD-5 hydrogel, while when the other amino acids (L-Ala, L-His, L-Cys, L-Phe, L-Gln, L-Ser, L-Thr, L-Asn, L-Val and Gly) were added, the fluorescence intensity changed slightly (Figure 6a–c), we then take L-Arg as an example to investigate in detail. With the increase of the concentration of L-Arg, the fluorescence intensity gradually decreases (Figure 6d), I_0_/I have a linear relationship with L-Arg at a lower concentration, and the detection line calculated from 3δ/slope is 240 μM (Figure 6e), indicating that Ag_9_-NCs/DD-5 hydrogel can detect L-Arg with high sensitivity. Adding L-Arg and other amino acids together to the hydrogel system, it could be found that even in the presence of other amino acids, L-Arg could still quench its fluorescence, indicating that Ag_9_-NCs/DD-5 hydrogel also has high selectivity for L-Arg (Figure 6c).

In addition, it was found from the TEM image that the nanotubes with a little spiral disappeared after the addition of L-Arg, and more particles with superfine nanowires appeared (Figure 6f), indicating that the addition of L-Arg destroyed the ordered structure of the hydrogel. From FT-IR results, as shown in Figure 6g, after the addition of L-Arg, there is a reappearance of peaks at 1658 and 1578 cm^−1^ belonging to the amide I band (its red shift is attributed to the addition of L-Arg) and at 1392 cm^−1^ belonging to the symmetric vibrational absorption peak of the C=O group in mba^2−^, indicating that the addition of L-Arg destroyed the intermolecular H-bond and reduce the radiation relaxation of the Ag_9_-NCs ligand mba^2−^ and made the fluorescence disappear. In the FT-IR, it is found that L-Arg is added to break the H-bond between Ag_9_-NCs and DD-5 to achieve the purpose of detection, which has nothing to do with the molecular configuration of Arg. Therefore, we reasonably believe that Ag_9_-NCs/DD-5 hydrogel has good detection capabilities for both L-Arg and D-Arg.

## 4. Conclusions

In this work, an Ag_9_-NCs/DD-5 hydrogel with highly ordered hollow nanotubes with a little spiral and phosphorescent emission was successfully obtained by supramolecular self-assembly through inter-ligand non-covalent interactions (H-bond, π–π stacking) and argentophilic interactions [Ag(I)–Ag(I)] between Ag_9_-NCs. After self-assembly, the transfer of chirality of DD-5 to supramolecular chirality of Ag_9_-NCs/DD-5 hydrogel was successfully realized together with good CPL performance. In addition, Ag_9_-NCs/DD-5 xerogel can still emit fluorescence at 200 °C, making it an ideal choice for a new generation of luminous temperature-sensing agents both in low and high temperatures. Moreover, Ag_9_-NCs/DD-5 hydrogel has selectivity and sensitivity for the detection of L-Arg and D-Arg. Our work implied that supramolecular self-assembled materials for metal NCs not only have potential applications in the field of fluorescence sensing, but also have huge application prospects in the field of biological detection.

## Data Availability

Not applicable.

## Supplementary Materials

The following supporting information can be downloaded at: https://www.mdpi.com/article/10.3390/nano12030424/s1, Figure S1: Co-assembled primitive photos and co-assembled phase behavior photos, Figure S2: TEM and SEM images at different concentrations of DD-5, Figure S3: PL decay curve of lyophilized Ag_9_-NCs solution and Ag_9_-NCs/DD-5 hydrogel, Figure S4: High-resolution XPS spectra of the Ag_9_-NCs/DD-5 xerogel, Figure S5: Hydrogel rheology and xerogel TGA, Table S1: Lifetime of the powder of lyophilized Ag_9_-NCs solution, Table S2. Lifetime of Ag_9_-NCs/DD-5 hydrogel.
