# Supplementary material for: Supramolecular Self-Assembly of Atomically Precise Silver Nanoclusters with Chiral Peptide for Temperature Sensing and Detection of Arginine
Source: Nanomaterials (Basel). 2022 Jan 27;12(3):424. doi: 10.3390/nano12030424 (PMC8839151; doi:10.3390/nano12030424)
Supplement: Supplementary file 1 [file nanomaterials-12-00424-s001.zip › nanomaterials-1545957-supplementary.pdf]

Supporting Information

Supramolecular Self-assembly of Atomically Precise Silver Nanoclusters with Chiral Peptide for Temperature Sensing and Detection of Arginine

Wenjuan Wang, Zhi Wang, Di Sun, Shulin Li, Quanhua Deng, Xia Xin*

National Engineering Research Center for Colloidal Materials, Key Laboratory of Colloid and Interface Chemistry (Ministry of Education), School of Chemistry and Chemical Engineering, Shandong University Jinan, 250100, China

---

*Author to whom correspondence should be addressed, E-mail: xinx@sdu.edu.cn

Phone: +86-531-88363597.

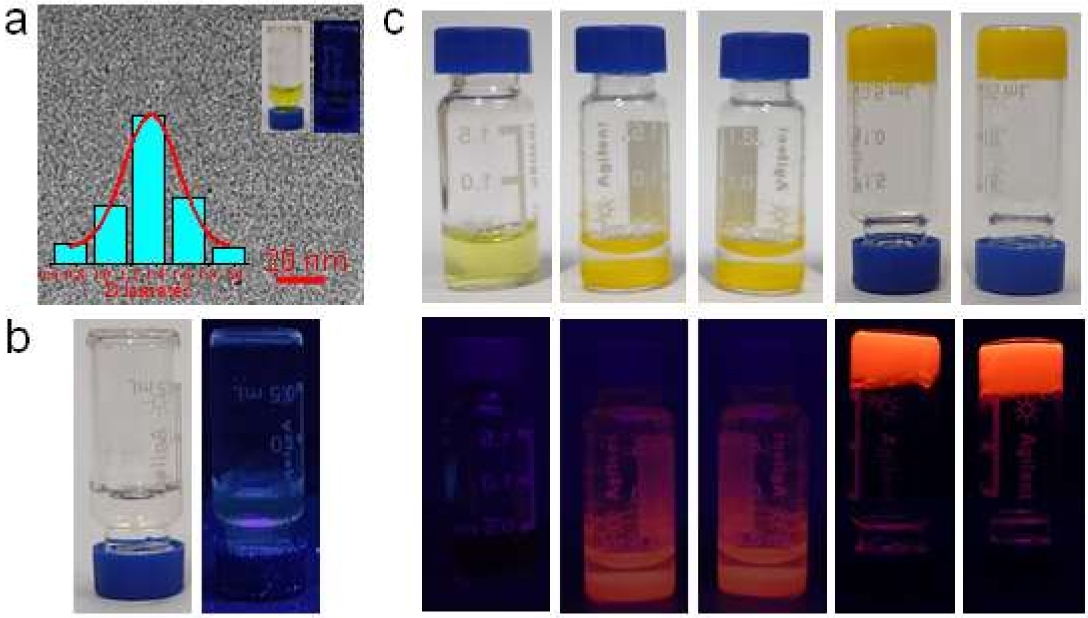

Figure S1. Co-assembled primitive photos and co-assembled phase behavior photos. (**a**) HR-TEM image of 5 mM Ag_9-NCs in aqueous solution, inset: the results of particle size distribution of Ag_9-NCs and photographs taken under daylight (left) and 365 nm UV light (right); (**b**) DD-5 photographs taken under daylight (left) and 365 nm UV light (right); (**c**) Phase behavior and photographs taken under daylight (up) and 365 nm UV light (down) of Ag_9-NCs at different DD-5 concentrations. (From left to right: 39 mM, 42 mM, 43 mM, 60 mM, 70 mM).

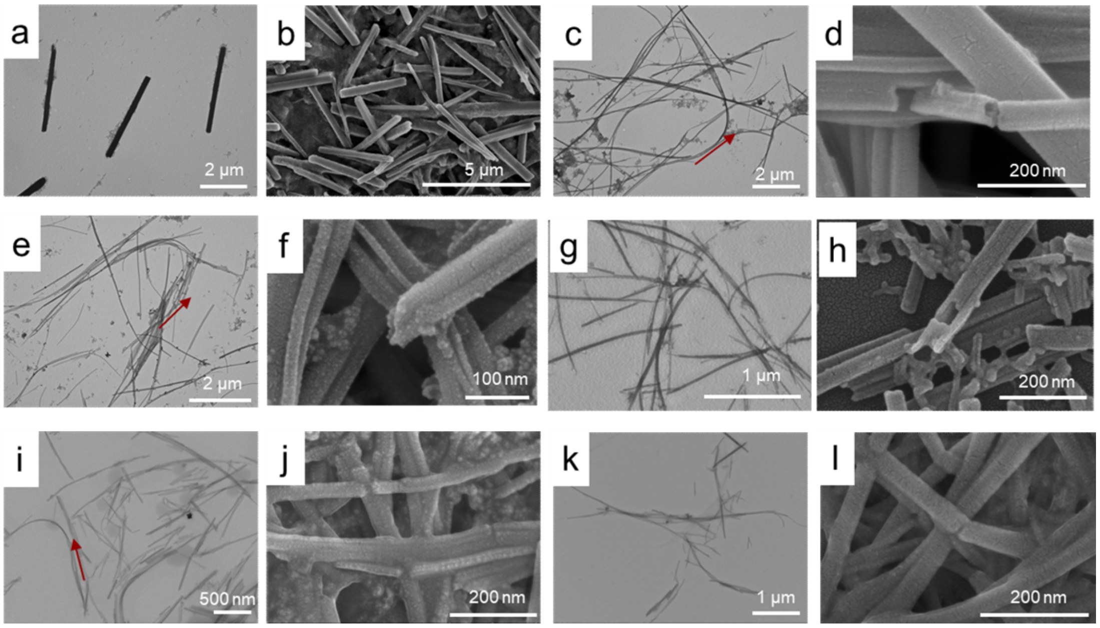

Figure S2. TEM and SEM images at different concentrations of DD-5. (**a, b**) 5 mM Ag_9-NCs/40 mM DD-5; (**c, d**) 5 mM Ag_9-NCs/50 mM DD-5; (**e, f**) 5 mM Ag_9-NCs/60 mM DD-5; (**g, h**) 5 mM Ag_9-NCs/70 mM DD-5; (**i, j**) 5 mM Ag_9-NCs/80 mM DD-5; (**k, l**) 5 mM Ag_9-NCs/90 mM DD-5.

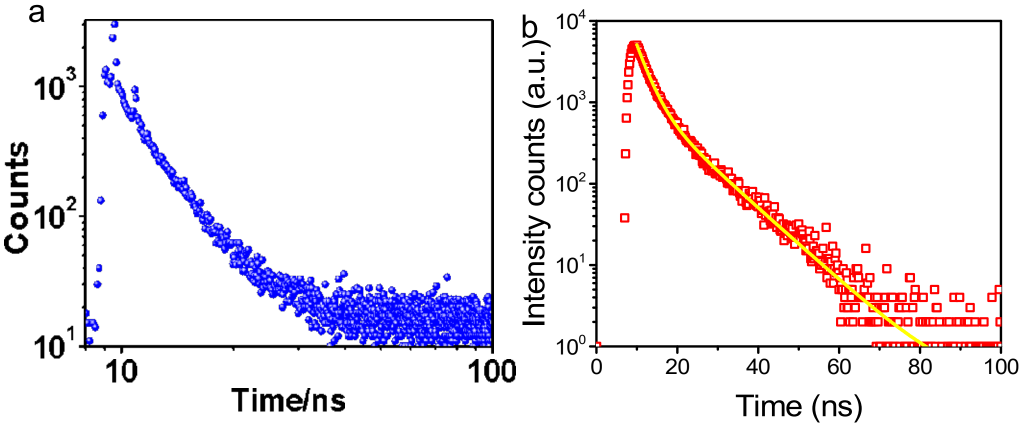

Figure S3. PL decay curve of lyophilized Ag_9-NCs solution and Ag_9-NCs/DD-5 hydrogel. (**a**) lyophilized Ag_9-NCs solution; (**b**) Ag_9-NCs/DD-5 hydrogel.

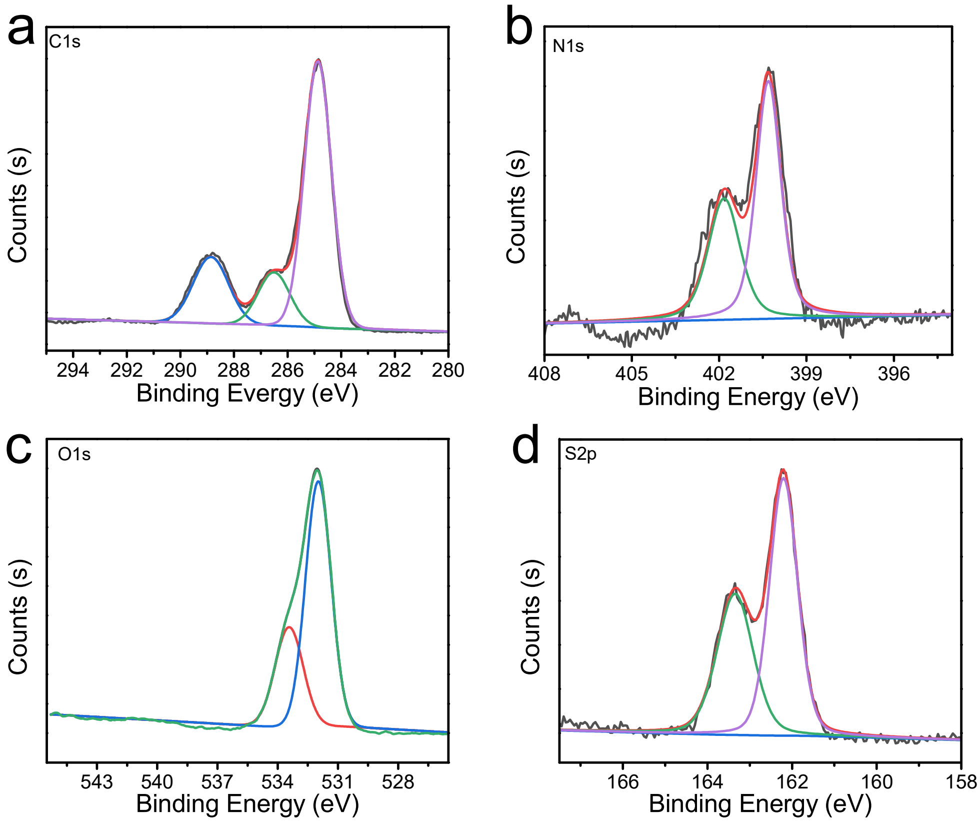

Figure S4. High-resolution XPS spectra of the Ag_9-NCs/DD-5 xerogel. (**a**) C_{1s}; (**b**) N_{1s}; (**c**) O_{1s} and (**d**) S_{2p}.

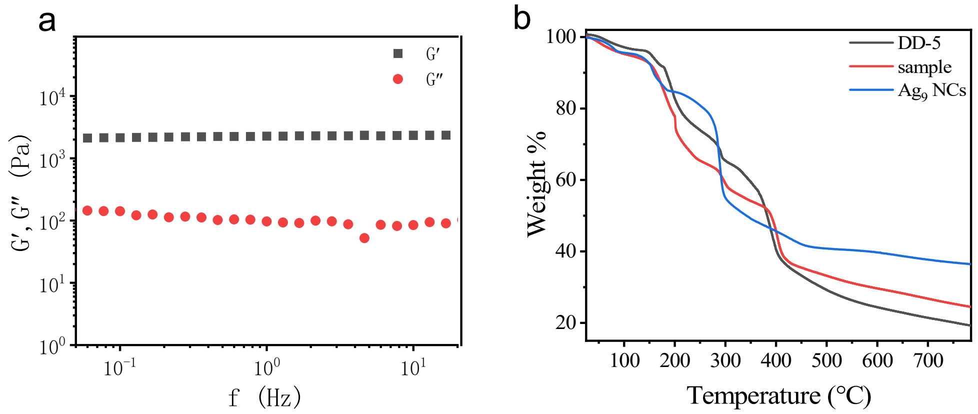

Figure S5. Hydrogel rheology and xerogel TGA. (**a**) frequency tests at τ = 10 Pa for the Ag_9-NCs/DD-5 hydrogel; (**b**) TGA of a lyophilized Ag_9-NCs solution, DD-5 and Ag_9-NCs/DD-5 xerogel.

Table S1. Lifetime of the powder of lyophilized Ag_9-NCs solution.

Sample/Lifetime	τ_2 / ns	τ_2 / ns	τ_{ave} / ns
Ag_9-NCs	0.032 (13.1%)	1.973 (50.7%)	3.277

Table S2. Lifetime of Ag_9-NCs/DD-5 hydrogel.

Sample/Lifetime	τ_2 / ns	τ_2 / ns	τ_{ave} / ns
hydrogel	2.7584 (51.89%)	9.7149 (48.11%)	6.105
